# Design and fabrication of complete dentures using CAD/CAM technology

**DOI:** 10.1097/MD.0000000000005435

**Published:** 2017-01-10

**Authors:** Weili Han, Yanfeng Li, Yue Zhang, Yuan lv, Ying Zhang, Ping Hu, Huanyue Liu, Zheng Ma, Yi Shen

**Affiliations:** a Department of Stomatology, First Affiliated Hospital of Chinese PLA General Hospital; b Department of Stomatology, Beijing Shen Yi Denture Processing Center, Beijing, China.

**Keywords:** 3D digital edentulous models, CAD, CAM, complete dentures, milling

## Abstract

The aim of the study was to test the feasibility of using commercially available computer-aided design and computer-aided manufacturing (CAD/CAM) technology including 3Shape Dental System 2013 trial version, WIELAND V2.0.049 and WIELAND ZENOTEC T1 milling machine to design and fabricate complete dentures.

The modeling process of full denture available in the trial version of 3Shape Dental System 2013 was used to design virtual complete dentures on the basis of 3-dimensional (3D) digital edentulous models generated from the physical models. The virtual complete dentures designed were exported to CAM software of WIELAND V2.0.049. A WIELAND ZENOTEC T1 milling machine controlled by the CAM software was used to fabricate physical dentitions and baseplates by milling acrylic resin composite plates. The physical dentitions were bonded to the corresponding baseplates to form the maxillary and mandibular complete dentures.

Virtual complete dentures were successfully designed using the software through several steps including generation of 3D digital edentulous models, model analysis, arrangement of artificial teeth, trimming relief area, and occlusal adjustment. Physical dentitions and baseplates were successfully fabricated according to the designed virtual complete dentures using milling machine controlled by a CAM software. Bonding physical dentitions to the corresponding baseplates generated the final physical complete dentures.

Our study demonstrated that complete dentures could be successfully designed and fabricated by using CAD/CAM.

## 1 Introduction

Edentulism has been a serious public health problem in industrialized countries due to population aging and in developing countries due to poor oral care.^[1]^ It has been estimated that 26% seniors in USA have edentulism, and edentulous population of seniors is 15% to 78% in Europe, 24% in Indonesia, 11% in China, and 23% in Brazil.^[2]^ The life quality and nutrition intake are impacted for edentulous patients due to edentulism.^[3]^ Complete dentures are 1 mainstay choice for edentulous patients.^[4,5]^ Despite an anticipated decrease in the age-specific rates of edentulism, the demand for complete dentures will continuously increase in the next decades.^[4–7]^

Currently, complete dentures are mainly designed and fabricated using conventional methods, which involve a broad series of clinical and laboratory procedures.^[8]^ To obtain complete dentures, edentulous patients typically have to make 5 visits to the dental clinics, including preliminary impressions, final impressions, recording jaw relations, trial placement of wax denture, and placement/insertion of complete dentures. These clinical and laboratory procedures are mainly performed manually. Therefore, it is very challenging to ensure the quality for the manually designed and fabricated dentures. Moreover, it is difficult to keep and re-use those physical models generated in the process to generate additional complete dentures latter when the patients need them.

Computer-aided design and computer-aided manufacturing (CAD/CAM) has emerged as a new approach for the design and fabrication of complete dentures.^[9,10]^ The use of CAD/CAM technology in the field of dentistry could be traced back to the early 1980s.^[11]^ However, unlike the extensive use of this new technology in the other aspects of dentistry, the use of CAD/CAM was limited in the production of complete dentures due to the lack of suitable CAD software until recently.^[4,9–17]^ Several commercial CAD software systems, including 3Shape Dental System and AvaDent digital dentures,^[14,18]^ have recently become available for designing complete dentures. With this CAD/CAM technology, only 2 appointments are needed for patients to get their complete dentures. All impressions, jaw relations, occlusal plane orientation, tooth mold and shade selection, and maxillary anterior tooth positioning could be finished in 1 patient visit for the fabrication of complete dentures, saving a lot of time and materials for both patients and/or dentists.

However, the reports on the use of CAD/CAM technology to produce complete dentures are still limited in the literature.^[9,10]^ In this study, we report our efforts to design and fabricate complete denture using commercially available CAD software and CAM-controlled milling machine. Based on 3D digital edentulous models generated by scanning the physical edentulous models, virtual complete dentures were designed using the modeling process of full denture available in the trial version of 3Shape Dental System 2013. The designed virtual complete dentures were exported to the CAM software of WIELAND V2.0.049. Physical dentitions and corresponding baseplates were fabricated by milling acrylic resin composite plates using WIELAND ZENOTEC T1 milling machine controlled by WIELAND V2.0.049 according to designed virtual complete dentures. Compete dentures were produced by bonding the physical dentitions to the corresponding baseplates.

## 2 Materials and methods

### 2.1 Materials and equipment

The equipment used in this study included 3Shape D810 scanner (accuracy: 15 μm. 3Shape, Denmark), DELL Optiplex 780 (Intel Core 2 i7, CPU 2.67 GHz, cache 4G RAM, memory 512MB) with the operation system of Windows 7 (64 bit) and ACD software of 3Shape Dental System 2013 trial version, and Dell Precision T1600 (i3 CPU, and cache 8G) connected with WIELAND ZENOTEC T1 (WIELAND, Germany) (accuracy: 10 μm) using Windows 7 as the operation system, and WIELAND V2.0.049 as the CAM software.

Acrylic composite resin plates (98 mm in diameter, and 25 mm or 16 mm in thickness) were purchased from WIELAND (Germany). Dental alginate impression material and die-stone were purchased from Heraeus (Germany).

All data were provided in full in the following sections of this paper.

### 2.2 Generation of physical edentulous master cast, and wax occlusal rims and baseplates

The study was approved by the Ethics Committee of First Affiliated Hospital of Chinese PLA General Hospital. Customized physical wax occlusal rims and baseplates, and master cast were obtained from a female edentulous patient with missing teeth for 30 years and order II alveolar ridge resorption (according to the classification proposed by Atwood,^[19]^ and Cawood and Howell^[20]^). Before the impression, the patient gave informed consent which was approved by the institutional review board (IRB). Alginate impression material was used to obtain the impression, after which the physical master cast, wax occlusal rims, and baseplates were produced using the traditional clinical method.^[9,11]^

Since 3Shape Dental System 2013 does not support the trimming of post dam area in the 3D digital edentulous models, we trimmed the post dam area on the physical master cast. Briefly, a line was drawn along the pterygomaxillary notches of both sides and the points at 2 mm behind the foveae palatinae on the master cast. A 1 to 1.5-mm deep cutting line was made using sculpt knife along the vibration line. A layer of cast was removed from the area covering this cutting line to 5 mm before this line. Less was removed if the place was further away from the cutting line. A bow shape was formed by gradually shallowing this area, and was in parallel to the mucosal surface of the palate.

### 2.3 Design of virtual complete dentures

**Figure 1:**
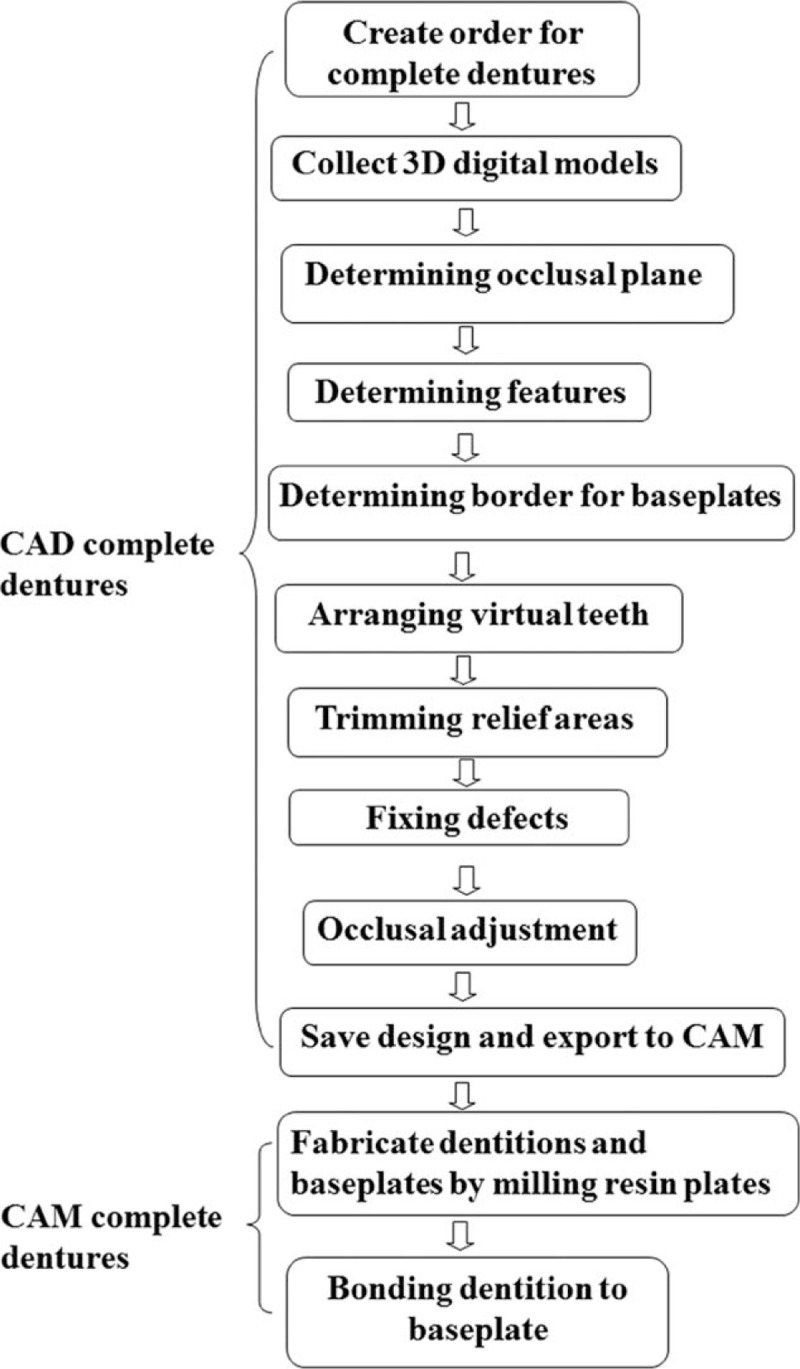
The flowchart for designing and fabricating complete dentures via CAD/CAM technology. CAD = computer-aided design, CAM = computer-aided manufacturing.

First we investigated the feasibility of designing virtual complete dentures starting from the physical models by using the modeling process of full denture available in 3Shape Dental System 2013. During the design, we generated 3D digital edentulous models, determined the occlusal plane, the feature points, and the boarder for baseplates, arranged artificial tooth dentitions, fixed local defects, and adjusted occlusal relation (Fig. 1). The details are described as the following.

#### 2.3.1 Creation of work order

In the 3Shape Dental System 2013, work order was created by selecting 17 to 27 and 37 to 47 artificial teeth for upper and lower dentitions, respectively, selecting the type of artificial teeth, selecting gingiva, and finishing the creation of upper and lower dentitions.

#### 2.3.2 Generation of 3D digital edentulous models

**Figure 2:**
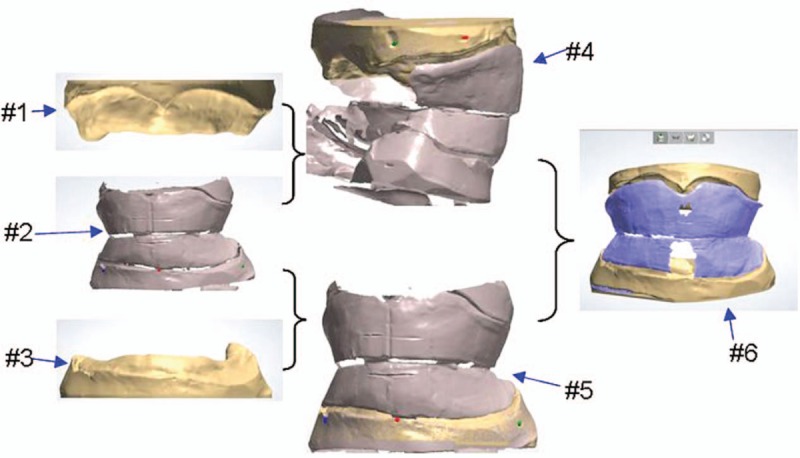
The process to generate 3D digital edentulous models for CAD, including separated 3D digital models of maxillary master cast (#1), mandibular master cast (#2), and maxillary occlusal rim with maxillary baseplate and mandibular occlusal rim with mandibular baseplate (#3). 3D = three-dimensional, CAD = computer-aided manufacturing.

The 3D noncontact laser scanner 3Shape D810 was used to scan the physical master cast and wax occlusal rims with baseplates to obtain 3 separated 3D digital models, including maxillary master cast (#1), mandibular master cast (#2), and maxillary occlusal rim with maxillary baseplate, and mandibular occlusal rim with mandibular baseplate (#3) (Fig. 2). Point-by-point matching approach was used to match model #1 and model #3, and model #2 and model #3, respectively, for obtaining models #4 and #5. The matching of models #4 and #5 generated model #6. After completing the matching process, complete 3D digital edentulous models were finally obtained for use in CAD with the relative position relationship of the maxillary and mandibular jaws (Fig. 2).

#### 2.3.3 Model analysis

The 3Shape Dental System 2013 supports the determination of occlusal plane for the 3D digital edentulous model. Three points were selected on the contacting plane of the upper occlusal trim and lower occlusal rim in the 3D digital edentulous model. The selection of these 3 points determined the occlusal plane for the digital edentulous model.

Feature points were selected at the maxillary tuberosity, center, and canine region of the maxillary arch, and the retromolar pad, center, and canine area of the mandibular arch. The margin lines of the baseplates were designed to meet the requirement for complete dentures and form the base for complete dentures. In particular, the labial and buccal ended at the mucobuccal fold between the alveolar mucosa and the labial and buccal mucosa, avoiding the labial/buccal frenulum; the lower jaw ended at the lingual frenulum, avoidinglingual frenulum, whereas the rear margin ended at the 1/3 to 1/2 of retromolar pad; the rear margin of the upper jaw ended at the line of pterygomaxillary notches of both sides and the points at 2 mm behind the foveae palatinae.

#### 2.3.4 Arrangement of artificial teeth

The teeth can be moved in the sagittal, horizontal, and coronal planes during the modeling using 3Shape Dental System 2013. The length, width, and height of dentition could be adjusted, and the localization of the dentition could be modified accordingly to match the arch shapes of the individuals. For each individual tooth, modification/adjustment could also be made for the whole tooth or the local regions of the tooth. Therefore, we chose a set of standard dentitions with A3 colored artificial teeth available in the Smile database for this study. All the available functions mentioned above were used to arrange the artificial teeth in the 3D virtual models to match the particular shapes of the patient's arches.

#### 2.3.5 Trimming relief area

**Figure 3:**
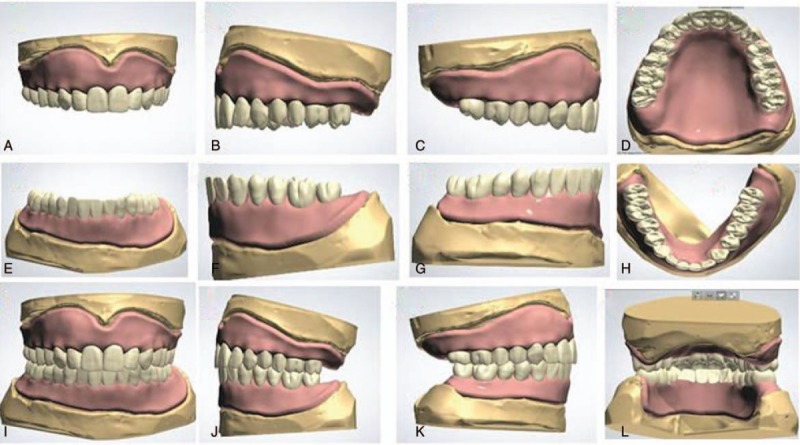
Different views of virtual complete dentures on the master cast: front view (A), view from the left (B), view from the right (C), and view from the occlusal plane (D) of virtual maxillary complete denture; front view (E), view from the left (F), view from the right (G), and view from the occlusal plane (H) of virtual mandibular complete denture; front view (I), view from the left (J), view from the right (K), and rear view (L) of complete dentures on master cast.

After finishing the arrangement of artificial teeth, relief was made for the maxillary zygomatic process, maxillary tuberosity, incisive papilla maxillary hard area, mandibular external oblique ridge, and the mylohyoid ridge relief area according to the requirements for complete denture. Thus, the designed virtual complete dentures had some local defects at the baseplates and gingiva. Complete dentures were obtained (Fig. 3) for the occlusal adjustment after fixing these local defects on the virtual baseplates and gingiva using sculpt and morphing tools available in 3Shape Dental System 2013.

#### 2.3.6 Occlusal adjustment

**Figure 4:**
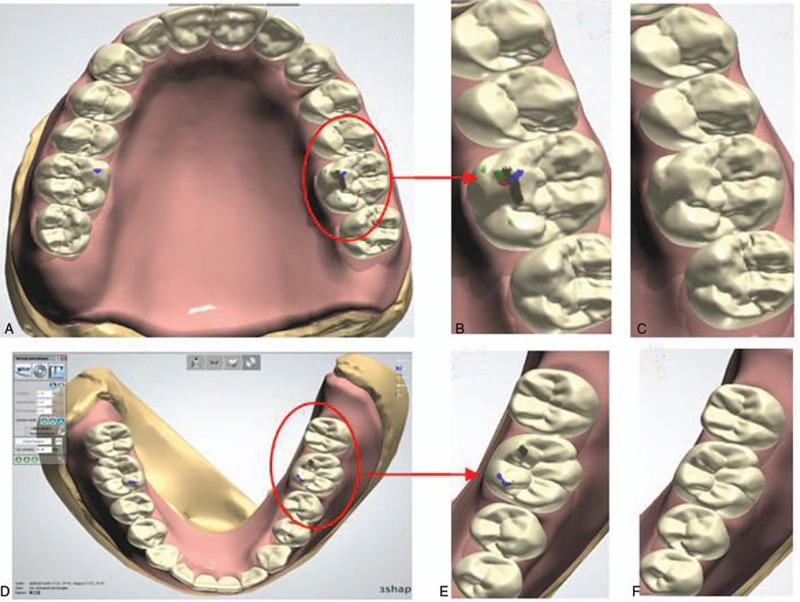
The detection of interfering points for virtual complete dentures and subsequent removal of interfering points: A, detected interfering points in virtual maxillary denture; B, the enlarged image of the red cycle in A; C, interference points removed from virtual maxillary denture; D, detected interfering points in virtual mandibular denture; E, the enlarged image of the red cycle in D; F, interference points removed from virtual mandibular denture.

The CAD software of 3Shape Dental System 2013 can mimic the movements of occlusion, laterotrusion, pro/retrusion, and side shift. Adjustable parameters for these virtual articulators include occlusal plane, Bennett L, Bennett R, Cond.incl.L, and Cond.incl.R. In this study, we specified 10° for Bennett, 34° for condylar guide, and −4 mm to +4 mm for side shift. During the virtual occlusal adjustment, the contacting points were recorded using different color for each single movement (Fig. 4A–D). Those interfering points were removed using the function of virtual grinding (Fig. 4C and E). The design of virtual 3D complete dentures was finished after completing this step of occlusal adjustment.

### 2.4 Fabrication of complete dentures

**Figure 5:**
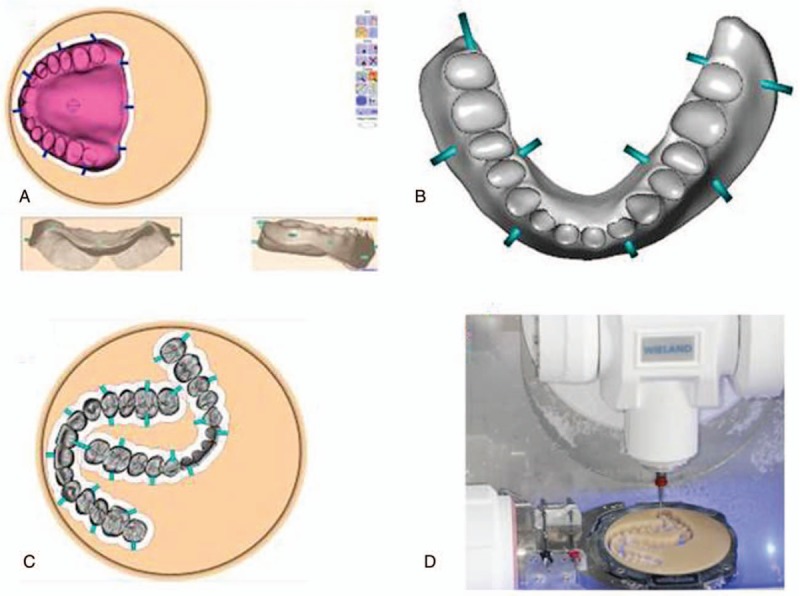
Fabrication of baseplates and dentitions using CAD technology, the virtual maxillary baseplate (A); the virtual maxillary baseplate (B); the virtual upper and lower dentitions (C); the milling of acrylic composite plate for upper and lower dentitions (D). CAD = computer-aided manufacturing.

The designed virtual complete dentures were exported to the CAM software of WIELAND V2.0.049 (Fig. 5A–C), which controlled a WIELAND ZENOTEC T1milling machine. The acrylic composite plates were milled into upper and lower baseplates, and upper and lower dentitions via the subtractive manufacturing using the WIELAND ZENOTEC T1milling machine (Fig. 5D). Tooth sockets were made on the baseplates for latter bonding the dentitions to the baseplates.

## 3 Results

**Figure 6:**
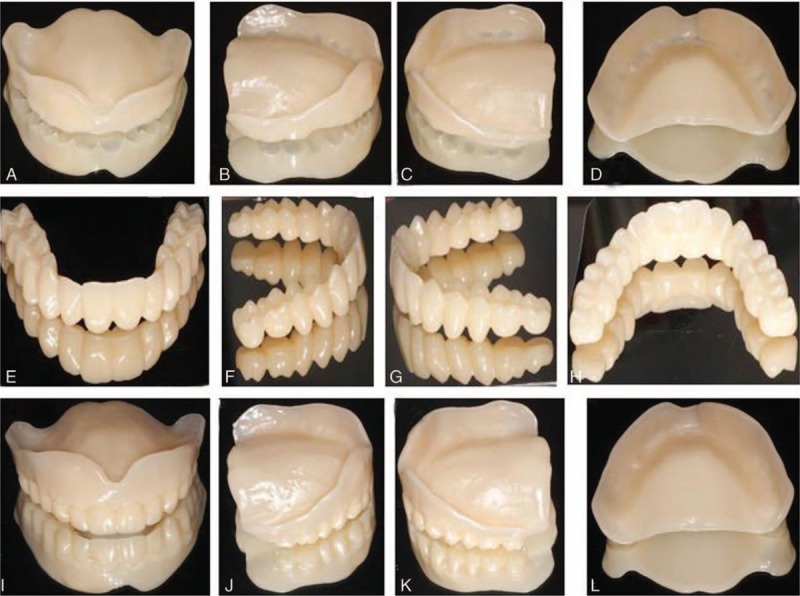
Fabricated maxillary baseplate: the front view (A), view from the right (B), view from the left (C), and rear view (D); maxillary dentition: the front view (E), view from the right (F), view from the left (G), and rear view (H); maxillary complete dentures: the front view (I), view from the right (J), view from the left (K), and rear view (L).

**Figure 7:**
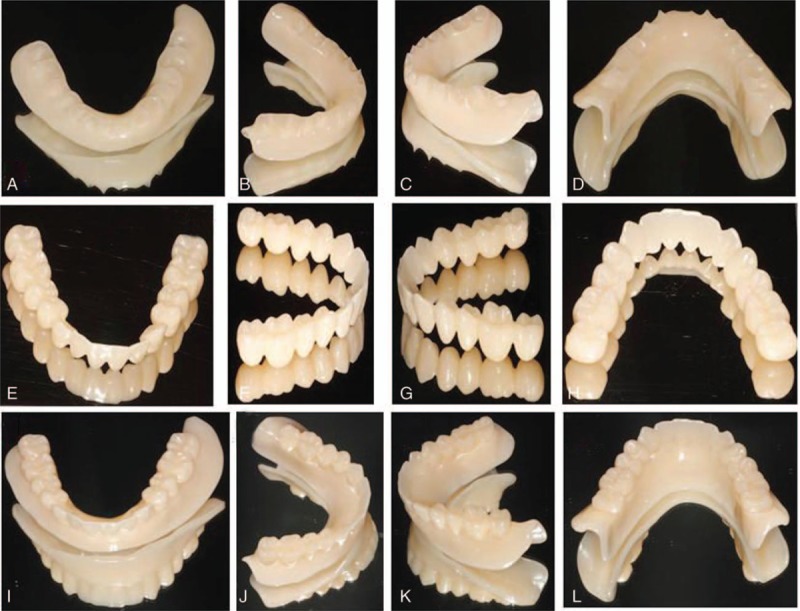
Fabricated mandibular baseplate: the front view (A), view from the right (B), view from the left (C), and rear view (D); mandibular dentition: the front view (E), view from the right (F), view from the left (G), and rear view (H); and mandibular complete dentures: the front view (I), view from the right (J), view from the left (K), and rear view (L).

The 3D digital edentulous models (Fig. 2) were first generated by scanning physical master cast, baseplates with occlusal trims, followed by point-to-point matching in 3Shape Dental System 2013. Based on these 3D digital edentulous models, virtual complete dentures (Fig. 3) were designed after several procedures for the first time using the trial version of 3Shape Dental System 2013. With the virtual complete dentures obtained after the occlusal adjustment, it took about 15 hours to fabricate the 2 baseplates (Figs. 6A–D and 7A–D) and 2 dentitions (Figs. 6E–H and 7E–H) by milling acrylic composite plates using WIELAND ZENOTEC T1 machine controlled by the CAM of WIELAND V2.0.049. The maxillary and mandibular complete dentures (Figs. 6I–L, and 7I–L) were obtained after bonding the upper dentition to the upper baseplate, and the lower dentition to the lower baseplate, respectively.

The produced complete dentures were successfully inserted into the patient's mouth. The patient was satisfied with the complete dentures during the use in terms of comfort and aesthetics.

## 4 Discussion

Maeda et al^[12]^ reported the pioneering work to design and fabricate complete dentures using CAD/CAM. They developed a computer-aided system including a work station for determining artificial tooth arrangements, occlusion, the outline of polished surface, and denture border location using a knowledge data base. A complete denture was fabricated from photopolymerized resin composite material using a 3D laser lithography machine via printing, but this complete denture had poor mechanical properties such as the strength, thus being unusable in patients.^[12]^ After this pioneering work, efforts have been made to develop computing programs or CAD software capable of designing complete dentures.^[4,21–24]^ However, the progress was slow to extensively exploit this CAD/CAM technology to fabricate complete dentures for edentulous patients due to the complicated construction for complete dentures, which significantly challenged the development of software or computing programs.

Recently, some systems of commercial CAD software have been improved to have the capability of designing complete dentures. We are among the very few pioneers to investigate the suitability of 1 such commercial system—the trial version of 3Shape Dental System 2013—for designing complete dentures. In this trial version, 1 more modeling process of full dentures was added over its 3Shape Dental System 2012. This newly added modeling process makes the design of complete denture easier than ever. The major steps involved are the generation of 3D digital edentulous models, model analysis (ie, determining the occlusal plane, feature points, and margin lines of denture bases), tooth arrangement, trimming relief areas, fixing local defects, and occlusal adjustment (Fig. 1).

The 3Shape Dental System 2013 supports the construction of 3D edentulous digital models via 3 scans for maxillary master cast, mandibular master cast, and maxillary occlusal rim with maxillary baseplate and mandibular occlusal rim with mandibular baseplate, and 2 steps of matching to match model #1 and model #3 for model #4, model #2 and model #3 for model #5, and consequent matching model #4 and model #5 for model #6, respectively (Fig. 2). The whole process could be finished within minutes.

The 3Shape Dental System 2013 has some significant improvements over the 2012 version. Unlike the 2012 version, the length and width of dentitions could be determined after specifying the feature points in mandibular and maxillary arches in the 2013 version. The occlusal plane for the dentitions remained the same as the preset occlusal plane, with only some fine tuning needed for minor adjustments. The upper and lower dentitions were correlated, which meant any change on 1 dentition would result in corresponding changes on the other, while maintaining the same occlusal relation. However, this might cause some problems when the anterior teeth need to be arranged for overbite. It is impossible to make such arrangement since the labial inclination would increase for the lower anterior teeth if the labial inclination of the upper anterior teeth is increased, resulting in normal occlusal relationship. We have forwarded our concerns on this issue to the 3Shape company for further improvement.

The other issue with the 2013 version is that although relieving the relief areas could be achieved on the 3D digital models, the modeling process does not support the trimming of the post dam area by reducing the height and area. Therefore, we had to complete this trimming on the physical model using the traditional methods. Virtual articulator can dynamically simulate the movement of individual mandibular jaw, and also simulate both static and dynamic occlusal contacts, thus providing more details than the oral and mechanical articulator could provide. The 3Shape Dental System 2013 software includes 5 famous digital articulators. It could simulate the occlusion, laterotrusion, pro/retrusion, and side shift through the customized parameter values input for the patients. In this study, we used the average values for the articulator to mimic the movements. Different colors were used to label the contacting zones during the different movement, with black for forward movement, blue for side shift, and red for Bennett movement. The interfering points were indicated by these colors on cusp tips (Fig. 4). In 3Shape Dental System 2013, these interfering points could be manually removed by using the function of “virtual grinding,” or automatically adjusted by the software based on the data recorded for the occlusal movements. However, these articulators lack connection with the trace recorder of the digital lower jaw's movements, and thus the movements could not be input into the virtual articulator.

The CAM technology could be classified into 2 types: additive manufacturing and subtractive manufacturing.^[10]^ The subtractive manufacturing relies on the computerized numerical control (CNC) machining.^[10]^ The desired geometry is obtained by physical removing the extra materials via machining (ie, cutting/milling) according to the digital model. The subtractive manufacturing has been extensively used to fabricate fixed prosthesis, such as inlays, onlays, veneers, crowns, and fixed bridges. Various materials such as composite resin, ceramic, titanium, and precious and nonprecious metals could be processed using subtractive manufacturing. In 2011, Kanazawa et al^[25]^ fabricated physical baseplates by milling acrylic resin plates via CNC according to the designed digital baseplates, following the design of complete denture using Dassault Systems (DassaultSystemes) from a 3D shape data generated by scanning patient's old complete dentures and standard artificial teeth. The use of preformed acrylic resin plates avoids the problems arising from polymerization shrinkage.

We adopted subtractive manufacturing method to fabricate the complete dentures (Fig. 5D). The designed virtual complete dentures had a thickness of 23.45 mm for upper baseplate, 20.08 mm for lower baseplate, 14.82 mm for upper dentition, and 12.20 mm for lower dentition, as determined using the CAM software. Currently, no multilayer composite resin blocks are commercially available for fabricating dentition and corresponding baseplate as 1 whole piece. Moreover, the resin composite block which could fit into the milling machine of WIELAND ZENOTEC T1 had to have a diameter of 98 mm and a maximum thickness no more than 25 mm. Thus, we fabricated baseplates and dentitions separately, and then bonded dentitions to the corresponding baseplates to form the complete dentures (Figs. 6 and 7). Each baseplate was fabricated from 1 resin plate (diameter 95 mm and thickness 16 mm), whereas the 2 dentitions were fabricated from the same resin plate (Fig. 5C) with the diameter of 95 mm and thickness of 25 mm. We attempted to find resin plates with similar color to the gingiva. However, the search for such resin plates was not successful and we had to use A3 colored acrylic composite resin for the fabrication of the designed baseplates. Thus, staining was necessary to match the color of baseplates fabricated to gingiva for better aesthetics.

For the fabricated dentitions and baseplates, we noticed that there were some gaps between some teeth and the corresponding denture bases. This might be attributed to the fact that the gingiva was designed to reach the high points of labial/buccal surfaces of the crowns for better morphology, resulting in some undercut areas. In addition, the accuracy of the machining instrument was affected by the long-term machining which generated too much heat. We resolved this issue by grinding the corresponding undercut areas. Moreover, we noticed some premature contacting points and occlusal interfering points in the produced complete dentures. In the design of virtual complete dentures, we had already performed the occlusal adjustment to remove the premature contacting and interfering points detected using the virtual articulators. The existence of premature contacting and interfering points in the produced complete dentures might be due to inaccurate removal of the detected points since the virtual articulators still lacked the capability of precise measurement and analysis. The other possible reason is that the accuracy of the machining instrument was affected by the long-term machining, which generated too much heat.

## 5 Conclusions

Our study demonstrated the suitability of design and fabrication of complete denture using the trial version of 3Shape Dental System 2013, the CAM of WIELAND V2.0.049, and WIELAND ZENOTEC T1 milling machine.
