# Design and fabrication of complete dentures using CAD/CAM technology: Erratum

**DOI:** 10.1097/MD.0000000000006030

**Published:** 2017-01-20

In the article “Design and fabrication of complete dentures using CAD/CAM technology”,^[1]^ which appeared in Volume 96, Issue 1 of *Medicine*, a funding statement was originally omitted. The funding statement should have read as follows:

This study was funded by the Clinical Support of PLA General Hospital (2014FC-SXYY-1003) and the Capital's Special Health Development Research (2014-4-5022).
